# Editorialets

**Published:** 1908-09

**Authors:** 


### Editorialets.

Bloodless Surgery as a Vaudeville Attraction.—The people of the Capital City of Texas are being amused and edified by the most unique and remarkable combination imaginable, of business and pleasure,—a vaudeville troupe, singers, dancers and actors, with a great surgeon as the *piece de resistance*. "Dr. La Fayette Berry" he calls himself; also, "The Phenomenal La Fayette." He is the exponent and apostle of bloodless surgery, and nightly, between acts, he removes scores of tumors, "kills" dozens of cancers, cures appendicitis while you wait—all without blood, pain, or the use of the knife. It is all duly written up in the Austin papers—and the addition made of the bushels and bushels of gall stones, 879 from one woman, it is claimed—"some of them as large as a marble"—without the use of the knife. This is not done in the presence of the multitude, however. Yes, multitude; for the people of all classes flock to see and hear him nightly—thousands! It is understood that he and his troupe are here (they have been here all summer) by invitation of the Austin Business League to amuse the people free of charge, and for some weeks he performed on the site of the old Capitol (State property) just at the Governor's front gate.

Dr. La Fayette has a license from the State Board of Examiners to practice medicine and surgery—bloody and bloodless—and is heeled. Who shall say him nay?

The *Austin Statesman*, September 9th, says:

"Fully 5000 people stood spellbound while La Fayette, the phenomenal exponent of bloodless surgery, removed a monster tumor from the head of O. B. Beard of Del Valle Saturday night on the platform where the doctor gives entertainments and lectures every night but Sunday.

"Mr. Beard has been troubled with these growths for many years, having a number of them upon his head. The one removed by the doctor Saturday night was very large, being very prominent and pronounced by physicians as dangerous. In the presence of these thousands of witnesses La Fayette began the operation. In a few moments the sac containing the monster was exposed, then with great rapidity the doctor performed without knife, anesthetic, pain or blood an operation which stamps him as a man of rare skill in his mighty profession of curing disease by the most modern methods. The tumor was entirely removed and Mr. Beard departed smiling.

"This is only one of the many cases where this phenomenal physician has successfully performed bloodless surgery. Appendicitis, gallstones, cancer are cured without the knife. Curable diseases yield to his marvelous skill. Many have been cured of diseases pronounced by others as incurable."

Alas! "Our noble profession!"

---

NOW IS THE TIME when the doctor has to get a move on and look after his collections. And now is the time when he should remember the "Red Back" and send in his little check for renewal of subscription and arrears.

---

"AND where was Moses buried?" asked the Sunday School teacher.

"On Mount Elbow," said Johnnie.

"Oh, no, Mount Nebo," said the teacher.

"Well, I knew it was one of the joints."

---

WANTED TO BUY A PRACTICE in Western Texas, if unopposed. Temperance community. Stock raising country preferred. Will also buy some property if suitable. Write full particulars as to roads, population, collections, price, etc., in first letter. Dr. J. L. G., care TEXAS MEDICAL JOURNAL, Austin, Texas.

---

THUMB SUCKING.—Johnnie, aged 5, persisted in sucking his thumb, despite scoldings and "lickings." Seeing the picture of the big-bellied man on the fences advertising Edgewood whiskey, he said, "Mommer, what made his stomach so big?" "Sucking his thumb," said his mother. A little later in a crowded street car Johnnie fixed his eyes on a young married lady of his acquaintance who had recently developed a bay window, and addressing her said with a knowing smile, "I know what *you* been doin'."

---

DISTINGUISHED GUESTS EXPECTED.—Dr. C. M. Rosser, of Dallas, Chairman of the Section on Surgery, Southern Surgical and Gynecological Association, has invited Dr. C. H. Mayo, of Rochester, Minn., and Dr. Alexander H. Ferguson, of Chicago, to be guests of that Association at Dallas on the occasion of its meeting in that city December 8th and 9th. Should they accept the invitation, due notice of the fact will be given and the presence of these great surgeons will attract a large attendance of Texas physicians.

---

GOOD OPENING.—A $3500 unopposed country practice in the richest black land farming belt in Texas, within twenty miles of Dallas, together with nice residence on large lot, orchard, outbuildings, office, drug store with $600 stock of drugs, can be bought for $1800. Advertiser will start purchaser to work on the spot, giving possession and turning over the practice to him. It can be made to pay for itself the first year. Not on railroad—five miles from station. Good reasons for the sacrifice. Address "Mack," care of the TEXAS MEDICAL JOURNAL, Austin, Texas.

---

PLATFORM DEMAND.—The State Democratic Convention incorporated in its platform "the enlargement and increase in scope and power of the public health department and better laws for the protection of the people against preventable diseases." This has been the hobby of the "Red Back" for twenty years. We may now, *possibly, get a* State Board of Health.

---

MISSISSIPPI VALLEY MEDICAL ASSOCIATION.—The thirty-fourth annual meeting of the Mississippi Valley Medical Association will be held in Louisville, Ky., October 13, 14, 15, 1908, under the presidency of Dr. Arthur R. Elliott, of Chicago.

Announcement has just been made of the selection of the orators for the coming meeting by the President. The Address in Medicine will be delivered by Dr. George Dock, Professor of Medicine in the University of Michigan, Ann Arbor; and the Address of Surgery by Dr. Arthur Dean Bevan, Professor of Surgery in Rush Medical College, Chicago. The mere mention of these names is enough of a warrant that this feature of the program will be in every way first-class.

The local Committee of Arrangements in Louisville has selected the Seelbach Hotel as headquarters, the general session and the section meetings being held in the hotel's large auditoriums.

One of the features of the entertainment projected is a smoker in the famous Rathskeller of the hotel—the finest of its kind.

The McDowell button, so much admired at the 1897 meeting in Louisville, will be reproduced in bronze for this meeting.

The members of the Mississippi Valley Medical Association, in attendance on the Louisville meeting, have been cordially invited by the management of the French Lick Hotel Company, French Lick, Ind., to stop over on Monday, October 12th, as guests of the hotel management.

Those who can accept this invitation are requested to notify the management, that proper provision for their entertainment can be made.

---

DIET IN DISEASES AND HEALTH.—By Cary H. Wilkinson, M. D., Galveston, Texas, who for thirty-three years was physician and surgeon to various hospitals, sanitariums and other institutions in the State of Texas. Dr. Wilkinson is in position to "speak as one having authority" in this matter. His little book—pocket size—contains twenty-five rules and formulas, covering every disease. It sells for 15 cents, and druggists will stock up on it if the doctor wishes to prescribe it for his patients. If not, order direct from the author. Dr. Wilkinson says:

THE ART OF FEEDING.—Equally important as medication and nursing stands the art of feeding in case of sickness. Few people understand this art, except as the fruit of lengthy training, with the result that, annually, thousands perish in this country from absolute starvation, even with a laden larder near at hand. In serving meals to invalids three cardinal rules are to be observed: First. Serve only *digestible food,* and such only as is suitable to the condition of the individual. Second. Serve often in small, or, at any rate, in *moderate quantities* at a time. Third. Serve everything in a *neat* and *attractive manner.* On these three rules depends the success in feeding in case of sickness. The object of this little book is to designate the most appropriate menu for particular ailments or conditions, as well as to point out, when necessary to do so, the amounts allowable, and the proper times at which foods for the invalid should be taken. To the general practitioner, hospital physician and trained nurse this book will be found indispensable, while in boarding schools and in private families it will prove a source of great convenience. It is not intended that every article on a diet list should be served at any one time, but only a *few selections* should be made, *alternately,* so that the invalid will be allowed a change in feeding every day. *Notate* the articles desired, or else *erase* the undesirable, and send list to the cook. There are 150 different selections to draw from in these diet lists.

---

### Ganderbone's Forecast for September.

BY C. H. RIETH.

Now Bryan was having
A speech phonographed,
When who should walk in
But the giant Bill Taft—
And they laughed.

"Good morning," said Bryan,
Displaying a look
Of joy and surprise
As he layed down his book—
And they shook.

"I have come for a visit,"
Said Taft, while his hat
Was hung on a nail
By the great Democrat—
And they sat.

"Delighted!" said Bryan,
"If I be allowed
The sentiment Ted
Has so often avowed"—
And he bowed.

"I suppose," ventured Taft,
With a smile that was sweet,
"You have just made a record
That I've got to beat"—
Very neat.

"Why no," Bryan laughed,
"I have records for two;
You may make one yourself
If you wish so to do—
After you."

But Taft waved his hand
With a show of suspicion,
And said, "I am not.
A machine politician"—
Intermission.

In the old Roman calendar September was the seventh month of the year. This brought Labor Day around in the heat of the summer. All the unions said it was too hot to march far enough to make any impression on capital. Like every other politician, Numa was afraid of the labor vote, so he pushed September along to the ninth place in the calendar and had Labor Day fall on the first day of autumn. The unions were thus enabled to march twice as far, and Numa had to refuse a third term.

The old school bell will toll the knell of youthful summer joys, and the girls will meekly get in line, together with some boys; but the gamer youngsters will hide out a few days in dissent, and later on the last one in will run for President.

The summer girl will get her coat and Merry Widow hat, and journey homeward from the sea uncertain where she's at; but the widow easily caressed because she stuck to toques, will bring a mollycoddle home and show it to the folks.

The tourists who have been abroad on fashionable trips will homeward wend with hotel tags stuck all around their grips; and thronging in their wake will come a never-ending flow of busted immigrants to see where they got all the dough.

September is when the autumnal equi*nox* the mosquito out. This occurs on the 22d, when the sun goes over the equator for a touchdown on the ice cream gigglery and summer underwear. The coal man will kick goal, and Mr. Roosevelt, leaving Sagamore Hill, will turn to Washington and the serious business of loading some more shells for lions.

The cooler air will stimulate
  The Presidential race,
And everybody will hit up
  A little faster pace.
The smiling entry from the Platte
  Will put up clouds of dust,
And the roly-poly man will run
  Till he is like to bust.

And Teddy meanwhile will observe the contest through his glass, and stick around the half-mile post until the runners pass; and if he fears the Platte will win the highest prized of boons, he'll laugh and whistle up a few old reassuring tunes, and toss a big fat bumblebee in Taft's back pantaloons.

And then there will be doing on this none too stable earth, and every Democrat will get his campaign dollar's worth; the Hisgens, Debs, and all of that consequential ray, will duck into the weeds and watch the big event go by; and the Taft men, looking on while the fireworks pop and sizz, will hold on tightly and enquire, "Which cloud of dust is his?"

The touch of fall will make the ripe
  And falling acorn thud,
And the crawfish will throw up his tail
  And burrow in the mud;
The dread mosquito will depart
  From this terrestrial scene,
But he'll die, as well becomes the brave
  With his face against the screen.

And then the fall-enlivened colt will frolic on the hill, and the railroads will return the folks they found too tough to kill; the fat and idle plutocrat will close his summer place, and the candidate will mount the stump and run off at the face.

After the 23d, September will be under the influence of Libra, the seventh sign of the zodiac. The sign of Libra represents a pair of scales held in the claws of a scorpion. It is of Chaldean origin, and is supposed to mean that about the 23d is where the ice man gets stung himself.

People born under Libra are incapable of pretense; the women never kiss women they hate, and the men play a wretched game of poker. Libra people also have no ear for music, and generally play some particularly loud instrument in the village band.

The swallow will desert the eave
  And start the movement south,
And the farmer prime himself to spit
  Through early autumn's drouth;
The pumpkins will grow long and gaunt
  With dragging on the vine,
And when the time shall come for old
  John D. to get in line
And pay his month's installment on
  That thirty million fine,
We'll hear a horse-laugh that will give
  Us shivers down the spine.

The moon will be full on the 10th, and the American fleet will make the Society Islands about the 12th. It is expected that it will remain there permanently, society having become its long suit.

With the advent of autumn, vice-presidential whiskers will begin to blend with nature's general color scheme, and both Mr. Sherman and Mr. Kern will run a little stronger on the tails of their tickets.

And then October will return,
That gladsome time and rare
When the pumpkin pie will answer "Here!"
Upon the bill of fare.

