# Correction

**Published:** 1908-10

**Authors:** 


CORRECTION.—The North Texas District Medical Society will meet in Dallas, December 8th and 9th, on which occasion the great surgeons, Mayo and Ferguson, will be the guests, instead of the Southern Surgical Association, as incorrectly stated in our last issue.

